# Using physical education to promote out-of school physical activity in lower secondary school students – a randomized controlled trial protocol

**DOI:** 10.1186/s12889-019-6478-x

**Published:** 2019-02-06

**Authors:** Juho Polet, Mary Hassandra, Taru Lintunen, Arto Laukkanen, Nelli Hankonen, Mirja Hirvensalo, Tuija Tammelin, Martin S. Hagger

**Affiliations:** 1 Faculty of Sport and Health Sciences, University of Jyväskylä, P.O. Box 35 (L335), 40014 Jyväskylä, Finland; 2 Faculty of Political Science, Helsinki, Finland; 3 LIKES Research Center for Physical Activity and Health, Jyväskylä, Finland; 4 School of Psychology, Curtin University, Perth, Australia

**Keywords:** Autonomy support, Behavioural intervention, Trans-contextual model, Self-determination theory, Theory of planned behaviour, Intervention development

## Abstract

**Background:**

Given the documented decline in levels of physical activity in early adolescence, promoting physical activity in young people is a priority for health promotion. School physical education (PE) is an important existing network in which participation in physical activity beyond school can be promoted to the captive young people. The objective of current article is to present the protocol for a PE teacher-delivered theory-based trial to promote secondary school students’ participation in physical activity out-of-school contexts. The intervention will be guided by the trans-contextual model explaining the processes by which PE teachers’ support for autonomous motivation in the classroom promotes students’ motivation to engage in out-of-school physical activity. We hypothesize that school students receiving the teacher-delivered intervention to promote autonomous motivation toward physical activity will exhibit greater participation in physical activities outside of school, relative to students receiving a control intervention.

**Methods:**

The trial will adopt a waitlist-control design with cluster-randomization by school. PE teachers assigned to the intervention condition will receive a two-week, 12-h training program comprising basic information on how to promote out-of-school physical activity and theory-based training on strategies to promote students’ autonomous motivation toward physical activity. Teachers assigned to the waitlist control condition will receive an alternative training on how to monitor physical functional capacity in children with special needs. PE teachers (*n* = 29) from eleven schools will apply the intervention program to students (*n* = 502) in PE classes for one month. Physical activity participation, the primary outcome variable, and psychological mediators from the trans-contextual model will be measured at pre-trial, post-trial, and at one-, three- and six-months post-trial. We will also assess teachers’ autonomy-supportive techniques and behaviours by observation.

**Discussion:**

The study will make a unique contribution to the literature by testing a theory-based intervention delivered by PE teachers to promote school students’ participation in out-of-school physical activity. Information will be useful for educators, community stakeholders and policy makers interested in developing programs to promote students’ out-of-school physical activity.

**Trial registration:**

ISRCTN39374060. Registered 19.7.2018.

**Electronic supplementary material:**

The online version of this article (10.1186/s12889-019-6478-x) contains supplementary material, which is available to authorized users.

## Background

### Promoting physical activity in school

Epidemiological data consistently indicate that levels of physical activity decline with age [1]. Consistent with these trends, national survey data from Finland indicate a decline in physical activity during adolescent years with only 41% of 11-year olds and 17% of 15-year olds meeting current national guidelines for physical activity [2]. Given that low levels of physical activity are related to increased risk of chronic illness later in life, and increased rates of conditions such as overweight and obesity [3, 4], the promotion of physical activity participation among young people is a public health priority. Physical education (PE) stands in an advantageous position for promoting the benefits of leisure physical activity as it addresses young, diverse and captive audiences [5]. Importantly, it is through PE that young people experience a variety of physical activities, and it is these experiences that may determine future involvement in physical activity during leisure time [6]. One of the primary aims of PE is to provide young people with the necessary motor skills, knowledge and competence to choose and participate in health-related physical activity in their leisure time [7]. Nevertheless, there is relatively little research outlining how PE teachers or PE programs can effectively orient young people toward participation in regular leisure-time physical activity outside of school.

The present article outlines the protocol of a trial in which PE teachers will be trained to support autonomous motivation toward leisure-time physical activity in lower-secondary school students (the PETALS trial). The trial aims to capitalize on school PE as an existing network to promote out-of-school physical activity in secondary school students. The trial will adopt a cluster randomized design and implement an intervention based on psychological theory to train participating teachers in techniques that support school students’ motivation to participate in physical activity in their leisure time outside of school. Trial effectiveness will be evaluated in terms of effects on participating school students’ post-intervention out-of-school physical activity participation. The theoretical basis for the intervention will be described next, followed by the study objectives.

### Theoretical basis for the intervention

The identification of factors that determine physical activity participation, and the processes by which they affect action, is paramount in providing formative evidence on which to base effective behavioural interventions [8]. The application of psychological theory, particularly theories of motivation and attitudes, has been at the forefront of providing an evidence base for the factors that drive participation in physical activity [9, 10]. However, only recently has this evidence has been applied to understand how teachers can promote students’ physical activity outside of school [10]. Such evidence is essential as it provides guidance on the content of interventions likely to be effective in promoting physical activity participation.

Self-determination theory is a prominent theory of motivation that has been applied to understand participation in health behaviours like physical activity [11, 12]. Central to the theory is the construct of self-determined or *autonomous* motivation. This form of motivation reflects an individuals’ general reflection on the causes of their action. Self-determined or autonomously-motivated individuals engage in actions such as physical activity out of interest, choice, and the sense of personal involvement they feel when engaged in the physical activities. In contrast, individuals who feel that their actions are less self-determined are likely to feel that their actions are controlled by external contingencies and engage in activities because they feel pressured, forced or obliged to do so. Research has indicated that autonomously-motivated individuals are more likely to persist with activities and more likely to gain positive or adaptive outcomes [13]. There is increasing research demonstrating that autonomous motivation is related to uptake and persistence with health behaviours [14] particularly physical activity [15, 16]. The proposed mechanism by which autonomous motivation leads to adaptive outcomes is through greater interest, effort, and involvement in the task [17].

Given that autonomous motivation has been shown to be related to persistence on adaptive behaviours, researchers and interventionists have sought to identify the contexts and conditions that promote and give rise to autonomous motivation. In particular, the focus on motivational ‘environment’ or ‘climate’ provided by the actions of significant others with leadership roles (e.g., coaches, teachers, instructors, bosses) has been shown to be influential in developing autonomous motivation. Specifically, leaders’ autonomy-supportive behaviours such as provision of choice and support for self-directed action have been shown to promote autonomous motivation and persistence in individuals operating in that environment [18, 19]. In addition, individuals’ perception that significant others in their environment are autonomy supportive have been shown to be strongly related to autonomous motivation as well as other adaptive outcomes in multiple contexts [14, 20–22]. In educational contexts, therefore, teachers can take the lead role in fostering autonomous motivation toward learning activities in the classroom by adopting autonomy supportive behaviours. Autonomy support focuses on style and delivery of lesson content rather than the content itself [23]. Interventions that have adopted autonomy support techniques and interactive styles have been found to be effective in multiple contexts in producing positive motivational and behavioural outcomes. Importantly, evidence exists that autonomy-supportive interventions can produce long-term changes in motivation and behaviour in academic settings [24, 25].

**Fig. 1:**
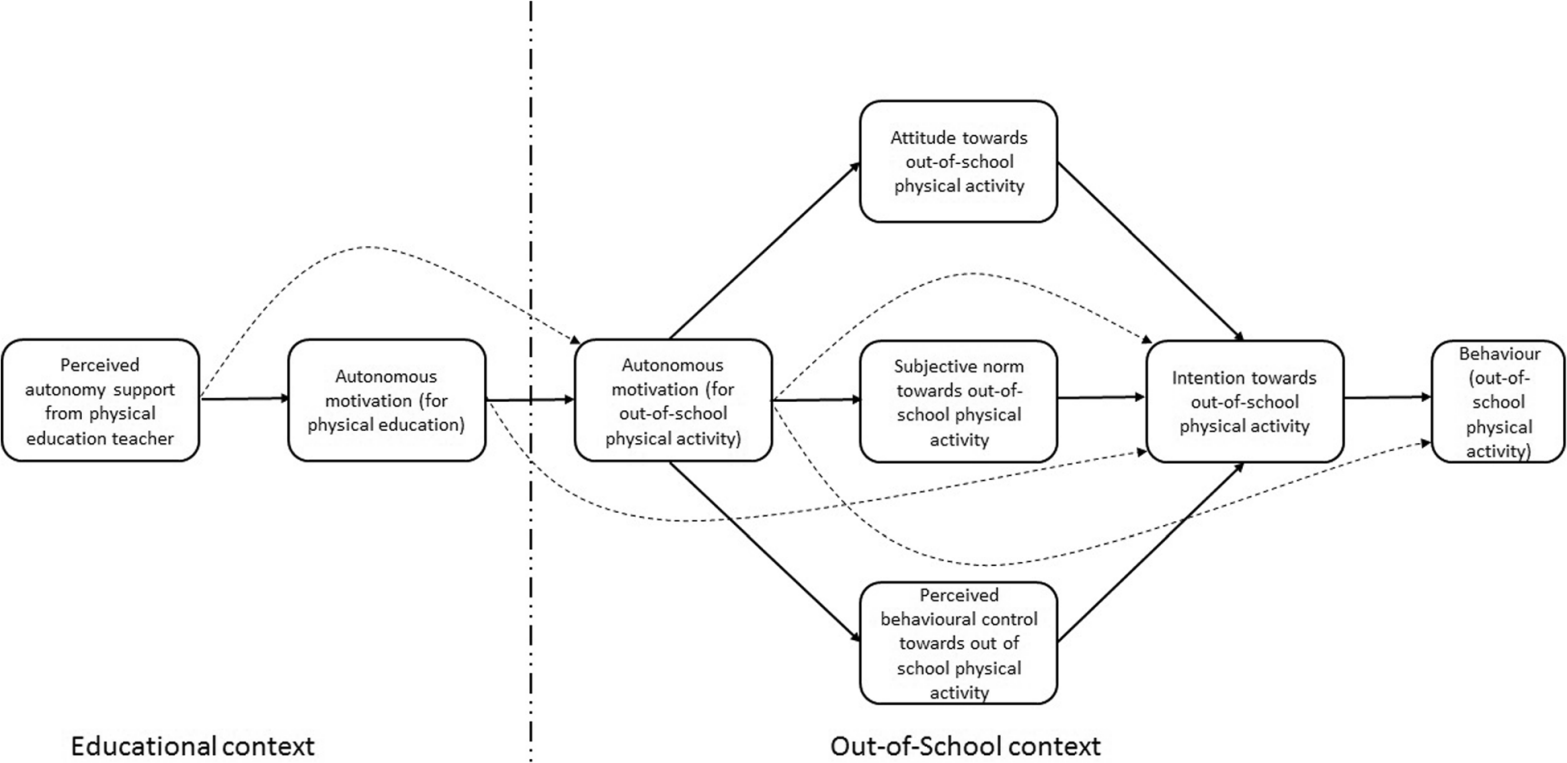
Theoretical framework: Trans-contextual model

While there is considerable research demonstrating links between the use of autonomy-supportive intervention techniques and student autonomous motivation and adaptive outcomes in class, comparatively less research has focused on the role that autonomy support in educational settings has on students’ behaviour outside the class (e.g., participation in sport and physical activities during leisure time). Recent theory has proposed the potential mechanisms by which autonomy support in an educational context like PE may lead to participation in physical activities outside of school. Capitalizing on multiple theories of motivation, particularly self-determination theory [12] and the theory of planned behavior [26] the trans-contextual model (TCM) was developed [6]. The model (Fig. 1) outlines how teachers’ autonomy support for in-class activities in PE context transfers to autonomous motivation toward, and future intentions to engage in, leisure-time physical activity in an out-of-school context. According to the model, teachers’ promotion of students’ autonomous motivation toward physical activities in PE will lead individuals to strategically align their motivation, beliefs, and intentions toward similar activities in related contexts with those motives. A review and meta-analysis of studies adopting the model provided support for model predictions across multiple studies [10]. In particular, the analysis supported links between autonomy support from teachers and autonomous motivation in school, consistent with previous research [27, 28]. In addition, the research supported trans-contextual links between autonomous motivation in school and autonomous motivation, beliefs (attitudes, subjective norms, and perceived behavioural control), and intentions toward participation in physical activity outside of school, and actual participation in physical activity outside of school.

The trans-contextual model provides a theoretical framework for developing interventions in educational contexts such as school PE to promote motivation toward, and actual participation in, related activities such as physical activity outside of school. The model implies that strategies aimed at fostering autonomous motivation will promote in-school and out-of-school autonomous motivation toward physical activity, and promote adaptive beliefs and intentions toward out-of-school physical activity and actual physical activity participation. These proposed effects are supported by research demonstrating the trans-contextual effects and confirming the relevant mechanisms involved [10]. The most effective means to support autonomous motivation in school is for teachers to display autonomy-supportive behaviours during PE lessons. Consistent with the model, effects of interventions aimed at promoting autonomy support in students on out-of-school physical activity is expected to be mediated by autonomous motivation in both contexts, beliefs, and intentions. Together, these mediators provide a demonstration of how the intervention functions in promoting physical activity behaviour. In other words, it provides a framework on *how* the intervention works.

The primary focus of this study is to test the effectiveness of an intervention based on the trans-contextual model in promoting out-of-school physical activity. However, we will also control for key demographic, environmental, and psychological variables that may moderate or affect intervention effects. With respect to demographic variables, we will control for effects of student’s age, gender, nationality, ethnicity and parental education level, given the potential for these variables to affect levels of physical activity and engagement in school. With respect to environmental variables, the autonomy support offered by parents and peers towards out-of-school physical activity, parental affection, and parental control may affect students’ performance and engagement in school [29, 30] and will also be considered covariates in our analysis of intervention effectiveness. Finally, we will also control for individual difference characteristics that have been shown to affect students’ motivation in previous research. These variables include grit [31] and self-discipline [32], two factors shown to be related to long-term effort and perseverance on tasks. Finally, we will also control for the extent to which students habitually perform physical activity out-of-school. The intervention may have less effect on students who have strong exercise habits, as they are already likely to exercise regularly and are unlikely to respond to motivational messages.

### Objectives

The purpose of the current protocol article is to report the development of a school-based intervention based on the trans-contextual model to promote secondary school students’ physical activity participation by fostering autonomous motivation (the PETALS trial). The trial will adopt a cluster-randomized waitlist-control design, and participants will be teachers of lower-secondary school PE classes and their students. The intervention will involve initial training of PE teachers of lower-secondary PE classes on the use of autonomy support strategies in their regular lessons, followed by an implementation period in which teachers apply their training in regular PE classes. Effects of the intervention will be evaluated through changes in subsequent follow-up measures of participating students’ physical activity levels and trans-contextual model variables relative to pre-trial baseline measures. We will also evaluate effects of the intervention on PE teachers’ autonomy-supportive behavior measured using self-report and observation. We will also control for support for autonomy from parents and peers. Other salient demographic and individual difference variables will be also controlled for. We expect the research will provide formative evidence of an effective, replicable, low-cost behavioural intervention, which will help in developing long-term participation in physical activity in young people. In addition, key deliverables of the research will be a set of training materials and an intervention manual, which will provide step-by-step accessible instructions on how to implement the intervention and can be disseminated to schools with no specialist knowledge and minimal cost.

## Methods

### Trial design

The study will adopt a cluster-randomized, wait-list control, single-arm intervention design with randomization by school. The trial comprises two phases: a teacher-training phase and an implementation phase. The teacher-training phase will comprise a two-week, 12-h training program in which secondary school teachers will receive the autonomy-support training program developed for the present study. The teacher-training program will be preceded by the pre-trial data collection occasion during which baseline measures of primary and secondary outcome variables will be taken. The training will be delivered by experienced teacher trainers as part of the teachers’ regular in-service training. The implementation phase will comprise a one-month period during which teachers will apply their training in their regular PE classes and it is followed by post-trial data collection occurrence. Thereafter, primary and secondary outcome variables will be collected at one-, three-, and six-month follow-up data collection occasions. Teachers allocated to the waitlist control condition receive a 4-h training program in which they will be instructed on how to apply a monitoring system for physical functional capacity in children with special needs [33]. Secondary school teachers (*N* = 29) from 11 secondary schools and their students (*N* = 502) in the city of Jyväskylä in central Finland will be invited to participate in the study.

### Participants and eligibility criteria

**Fig. 2:**
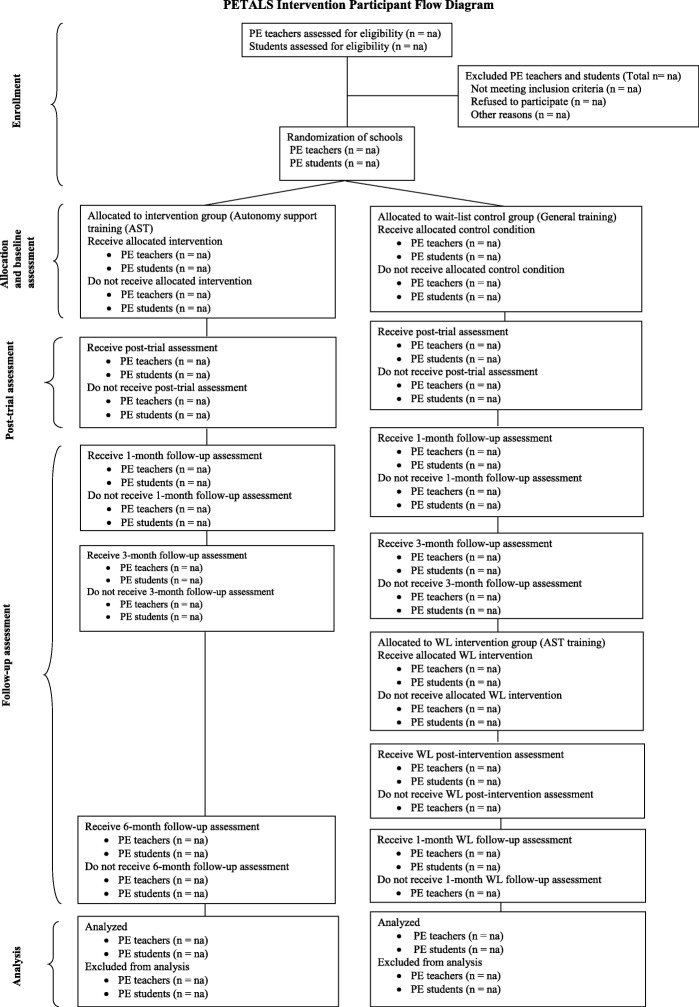
Participant flow diagram

Qualified full-time PE teachers teaching regular PE lessons in lower secondary schools will be eligible to participate in the study. Participating teachers will be asked to select one of their PE classes to be invited to participate in the study. Students in grades 7–9 (typical ages 13–15 years) in lower secondary schools will be eligible to participate. Students with existing physical or mental health condition that prevents participation in PE lessons, regular physical activity or completing surveys will be excluded. The proposed participant flow diagram through the trial is presented in Fig. 2.

### Recruitment process and informed consent

All available lower secondary level school PE teachers in the city will participate in the teacher-training phase of the study, irrespective of their participation, as the city Education Department has accepted the teacher-training phase to be part of PE teachers’ regular in-service training. We will recruit PE teachers and their students for the study via established links with schools and with support from the Education Department. Initial contact will be made with the head teacher of the school provided with details of the study aims and methods and the commitment required by the school. Once head teacher has consented their school to participate in the study, eligible teachers from each school will be invited to participate and provided with information on the study, and the benefits and requirements of participation, and given the opportunity to ask questions. Teachers agreeing to participate to the study will complete an opt-in informed consent form. Students of the PE teachers will be recruited to the study by referral from their teacher. Invitation letters, study information, and opt-out consent forms, with the exception of opt-in consent form for participation in the accelorometry component of the study measures, will be sent to eligible students’ parents or legal guardians via the schools’ online administration and communication software or via email or post. Students whose parents or guardians decline to give consent for their child to participate in the study will be exempted, and will be provided with alternative activities while participating students complete study measures at data collection time points.

### Procedure and data collection methods

The pre-trial baseline data collection will be scheduled for the third week after the beginning of the 2018–2019 school year. The following data will be collected: questionnaires administered to participating teachers and students comprising self-report measures, a one-week physical activity surveillance for participating students using accelerometers, and audio recordings of a selected PE class of each participating PE teacher. At pre-trial, all consenting teachers and students will complete a questionnaire containing demographic, psychological, and behavioural measures. Audio recordings of participating teachers’ classes will also take place during the baseline data collection. Physical activity behaviour will be collected from a subsample of students from the intervention and wait-list control groups using accelerometers for the week after the pre-trial data collection occasion. In addition, parents or legal guardians of participating students will complete self-report measures of demographic information, provision of autonomy support towards out-of-school physical activity, parental affection, and parental control they provide for their children at the baseline data collection.

Pre-trial data collection will be followed, consecutively, by the teacher training and implementation phases of the trial. In the teacher training phase, teachers allocated to the intervention group will receive the autonomy-support training program and teachers allocated to the wait-list control group will receive control education program over the same period. The completion of the training program will be followed by a 1 month implementation phase. In this phase, teachers in the intervention group will apply the techniques they learned in the training program in their regular PE classes.

**Fig. 3:**
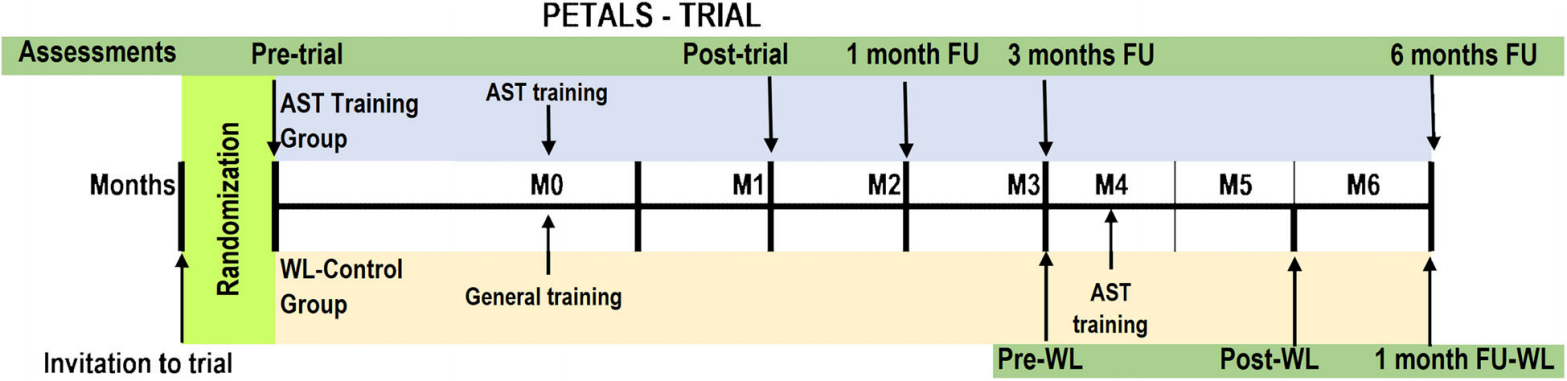
Timeline for data collection in months and participant contacts by group

Following the implementation phase, a post-trial data collection occasion will be scheduled. Data collection will comprise administration of measures identical to those at pre-trial with the exception of the baseline measures of behavioural automaticity, grit, self-discipline, parental affection, parental control, parental autonomy support and demographic measures. Accelerometer data and audio recordings of teachers’ lessons will also be collected from the same subsample of students and teachers, respectively. Follow-up data collection occasions are scheduled for one-, three-, and six-months post-trial. Accelerometer data from the subsample of students and audio recordings of teachers’ lessons will not be collected on the one-month follow up occasion. All assessments will be completed at the three- and six-month follow up data collection occasions, identical to the post-trial data collection occasion. Immediately after the three-month follow up data collection occasion, the wait-list control group will receive the autonomy-support training program. Post-trial and one-month follow up assessments will be conducted for teachers and students from this group, using identical measures as those administered in the intervention group (Fig. 3). Retention of participants will be maximized through pro-active email and telephone communication with school teachers who will provide access to students. Where collection of data on any given collection occasion is prevented due to unforeseen circumstances, we will negotiate an alternative occasion for the data collection as close as possible to the scheduled occasion.

### Intervention

#### Autonomy support intervention group

Teachers in schools allocated to the intervention condition will receive the twelve-hour interactive autonomy support teacher-training program developed specifically for this study. The program aims to familiarize PE teachers with techniques and strategies aimed at promoting students autonomous motivation toward out-of-school activities. The program focuses on six sets of autonomy-supportive strategies and techniques: Taking students’ perspective, using non-controlling and informational language, providing a rationale, displaying patience, providing choices, and accepting negative emotions and feelings. The techniques are adapted from the strategies identified in previous autonomy support training programs [34–37]. The training will be delivered by two trained teacher trainers with extensive experience of PE and teacher education. The trainers will undergo a familiarization session with the core research team in which they will be introduced to the training material (manual, power point presentations, supportive material) and provided with instruction on how to deliver the program prior to implementation of the training. A summary of session content and related delivery techniques are presented in Table 1.

**Table 1:** Description of teacher training program: Content and matched behaviour change techniques for each session

Session topic	Content	Behaviour change techniques^a^
1. Introduction and added value of the training to teaching practice	Introduction and warm up activities Information on the added value of the training and expectations Explore current supportive style and reflection Why autonomy support matters Introduction to self-determination theory	Social support (unspecified) Social support (practical) Discrepancy between current behaviour and goal Shaping knowledge Information about social and environmental consequences Imaginary reward
2. Autonomy supportive techniques: Description and benefits for students and teachers	Basics of the autonomy supportive teaching techniques: Definitions and implementation examples Using autonomy supportive techniques: Benefits for students and teachers based on previous research results	Demonstration of the behaviour Shaping knowledge Information about social and environmental consequences Behavioural practice/rehearsal
3. Use of autonomy supportive techniques to provide instructions	How, when, and why to use autonomy supportive techniques when giving instructions (organizational, technical and tactical) Taking students’ perspective Using non-controlling and informational language Providing rationale Providing choices Displaying patience	Information about social and environmental consequences Instruction on how to perform a behaviour Behaviour substitution Habit reversal Framing/ reframing Self-monitoring of behaviour Behavioural experiments Behavioural practice/rehearsal Graded tasks
4. Use of autonomy supportive techniques to provide feedback, encouragement, and praise	How, when, and why to use autonomy supportive techniques when providing feedback, encouragement, and praise Using non-controlling and informational language Taking students’ perspective Displaying patience	Generalization of a target behaviour Behaviour substitution Habit reversal Framing/ reframing Information about social and environmental consequences Instruction on how to perform a behaviour Self-monitoring of behaviour Behavioural experiments Behavioural practice/rehearsal Graded tasks
5. Use of autonomy supportive techniques to deal with discipline issues and off-task behaviours	How, when, and why to use autonomy supportive techniques when dealing with discipline issues and off task behaviours Taking students perspective Accepting negative affect Providing rationale Using non-controlling and informational language Displaying patience Providing choices	
6. Building personalized action plans	Plan changes in own teaching practice: Specific goals and plans for change when giving instructions, provide feedback, and respond to students with low motivation Identify barriers and problem solving for using the autonomy supportive techniques in every day teaching practice Development of their individualized infographic poster	Generalization of a target behaviour Goal setting Action planning (and implementation intention) Instruction on how to perform a behaviour Prompts/cues Adding objects to the environment Pros and cons Social support unspecified

^a^ From behaviour change technique taxonomy (Version 1) [66]

The teacher training was developed in three stages. In the first stage, we identified the most effective autonomy supportive techniques and means to deliver them to PE teachers based on current evidence. We therefore reviewed the existing literature on autonomy support techniques and training programs. Previous successful applications of autonomy support interventions in general classroom [38] and PE settings [39], as well as feasibility [36] and conceptual articles on autonomy support [34, 40] were identified. In addition, we acquired teacher training material from existing autonomy support training programs used in previous interventions [35, 37]. Next, we developed a list of training activities and delivery techniques considered effective and relevant to the proposed intervention. We also conducted a mapping exercise guided by a recent study outlining self-determination theory techniques and constructs [23] to ensure that the training activities and delivery techniques precisely matched the theory-based motivational determinants (e.g., autonomous motivation, psychological need satisfaction) targeted in the intervention.

The second stage involved the development of a detailed draft of the teacher training program. For each session, we developed a detailed description of the program content including aims, learning outcomes, main instructional points, examples, and interactive activities. Accompanying supportive materials (worksheets, printed examples, video demonstrations, presentation slides, and session’s summaries) were produced for illustration of the program content. The content and materials were reviewed and revised by the core research team.

The third and final stage involved review and revision of the entire program and materials by external stakeholders and teacher educators. Reviewers were experienced PE teachers and teacher-training experts, and researchers with expertise in the theoretical approaches on which the program was based, the delivery techniques used, and behavioural interventions conducted in school settings*.* The stakeholders reviewed each session content in detail in an interactive workshop with the research team. They also provided written feedback on the materials separately. Stakeholders identified issues relating to the clarity of the aims and descriptions, relevance of the examples, and overlap and redundancy in the materials. The program content and materials were further revised by the research team resulting in a final autonomy support training program with supporting materials.^1^

#### Wait-list control group

Participating teachers allocated to the waitlist control group will receive an alternative training program comprising 4-h of education on how to apply a monitoring system for physical functional capacity for children with special needs [33]. The control intervention is delivered in a one-day workshop by two educators experienced in PE teacher training.

### Outcome measures

All self-report outcome measures were translated from English into Finnish using a back-translation process by two bilingual researcher [41].

#### Primary outcome variable

The primary outcome measure is students’ post -intervention participation in out-of-school physical activity at the pre-trial, post-trial, one-, three and 6 month follow-up data collection occasions. Physical activity participation will be measured using the short form of the International Physical Activity Questionnaire, IPAQ [42], which will be modified to make explicit reference to out-of-school physical activity. The IPAQ comprises four items recording the frequency (number of days) and duration (hours) of participation in moderate and vigorous physical activity, walking, and sitting over the past 7 days. The physical activity score for moderate and vigorous physical activity and walking is calculated based on norms and expressed in MET-minutes per week. A score for total physical activity in out-of-school contexts is provided by the sum of the duration and frequency of vigorous, moderate and light physical activity scores. The IPAQ has acceptable concurrent validity and reliability indices [43].

#### Secondary outcome variables

Physical activity behaviour. A subsample of participants (approx. 120) from representative school classes covering grades 7–9 will have their physical activity participation measured using accelerometry. The purpose of this secondary measure is to provide concurrent validity and comparison data to support the IPAQ used as the primary outcome measure.^2^ Participants will wear the accelerometers (Hookie AM 20) for seven consecutive days after each data collection occasions at pre-trial, post-trial, and at the three-, and six-month follow up data collection occasions. These accelerometers have been shown to be valid and reliable in as a measure of physical activity in previous research [2]. Participants will be also asked to complete a short diary of their daily in-school and out-of-school physical activities for the period during which they wear the accelerometer. Data will provide duration participants spent in sedentary, light, moderate, and vigorous intensity physical activity per day. Diary data will be used to identify duration of physical activity on in-school and out-of-school contexts. It will also provide a measure of total energy expenditure in each context. For the purposes of the current study, we will compute total time spent in light, moderate, or vigorous physical activity and total energy expenditure in out-of-school contexts as criterion measure to test the concurrent validity of the IPAQ measure in each participant. The accelerometry data will be included only for those participants who have provided valid accelerometer data for a minimum of 3 days.

#### Mediating variables

All students will complete a battery of self-report measures of psychological variables based on the trans-contextual model. These factors are expected to reflect the mechanisms by which the intervention affects change in the primary outcome consistent with the model.

##### Students’ perceived autonomy support by their PE teacher

Perceived autonomy support from PE teacher will be measured using items from the perceived autonomy support scale for exercise settings [44]. The scale consists of 18 items (e.g., “I feel that my PE teacher provides me with choices and options to …”) and responses are provided on 7-point scales (1 = *strongly disagree* and 7 = *strongly agree*). The scale has demonstrated adequate construct validity and reliability statistics in previous research [44, 45].

##### Autonomous motivation, controlled motivation and amotivation toward in school and out-of-school physical activity

Autonomous and controlled forms of motivation for in-school and out-of-school activities will be measured using a modified version of the perceived locus of causality questionnaire [46], and amotivation using modified version of amotivation subscale from the sport motivation scale [47]. The total scale consists of ten items with two items measuring each of the external regulation (e.g., “I do PE/ physical activity so that the teacher won’t yell at me”), introjected regulation (e.g., “I do PE/physical activity because I would feel bad if the teacher thought that I was not good at PE”), identified regulation (e.g., “I do PE/physical activity because it is important to me to do well in PE/physical activity”), intrinsic regulation (e.g., “I do PE/physical activity because it is fun”) and amotivation (e.g., I do PE/ physical activity but I ask myself why I do it) constructs. Responses will be provided on 7-point scales (1 = *not true for me* and 7 = very true for me). For each of the PE and out-of-school contexts, autonomous motivation scores will be computed as an average of scores on the identified regulation and intrinsic regulation items, and controlled motivation scores will be computed as an average of scores on the external regulation and introjected regulation items. Amotivation will be measured with responses provided on the same seven-point scales. Measures for autonomous and controlled motivation have demonstrated satisfactory construct validity and internal consistency statistics in previous studies [45] and measure for amotivation has demonstrated adequate level of internal consistency [47].

##### Attitudes, subjective norms, perceived behavioural control, and intentions

Students’ attitudes, subjective norms, perceived behavioural control, and intentions with respect to their future participation in physical activity will be measured using scales developed according to reported guidelines [48]. Attitudes will be measured on three items in response to a common stem: “Participating in physical activity in the next month will be…” with responses made on seven-point scales (1 = *unenjoyable* and 7 = *enjoyable*). Subjective norms (e.g., “Most people who are important to me think I should do active sports and/or vigorous physical activities during my leisure time in the next month”), perceived behavioural control (“I am in complete control over participating in physical activity in the next month”), and intentions (“I intend to do active sports and/or vigorous physical activities during my leisure time in the next month”) will be measured on two items each with responses provided on seven-point scales (1 = *strongly disagree* and 7 = *strongly agree*). Previous research has supported the construct validity and internal consistency of these measures in the context of the trans-contextual model [45].

#### Additional measures

##### Observation of teacher autonomy supportive behaviours

Teacher’s use of autonomy-supportive behaviours in their lessons will be assessed using the tool for observing autonomy-supportive behaviours in teachers (TOAST) developed specifically for this study. The tool is a modified and extended version of checklist [49] for rating teachers’ autonomy-supportive and controlling behaviours in classroom contexts. The tool was augmented to include additional content based on the list of autonomy supportive and controlling behaviours identified in previous research [18]. The checklist was also developed to closely correspond to the autonomy supportive behaviours and strategies targeted in the autonomy support training program. The tool comprises three main categories of teacher behaviour: providing instructions, praise and encouragement, and dealing with misbehaviour. Each category is coded as autonomy supportive or controlling. Two additional categories, links with out-of-school physical activity and provision of an explanation or rationale, are coded as autonomy supportive only. The tool requires observers to note the frequency of behaviours displayed by the observed teacher in each category. Overall autonomy supportive and controlling behaviours in the first three categories, and autonomy supportive behaviours in the final two categories, are calculated by summing the frequencies of the observed behaviours in each category over the observation period. The open-source BORIS software is used for coding observations [50]. Research assistants blind to the purpose of the study will be trained by project researchers to code of the audio recordings from the lessons of participating teachers’ at baseline and at the scheduled follow-up data collection occurrences.

##### Behavioural automaticity

Behavioural automaticity, an important component of habit, will be measured using the four-item self-report behavioural automaticity index [51] (e.g., “Physical activity is something I do without thinking”, with responses provided on five-point scales (1 = *completely disagree* to 5 = *completely agree*). This scale has demonstrated satisfactory reliability and validity in previous research [51].

##### Grit

Student’s grit, defined as self-rated trait-level perseverance and passion for long-term goals, will be measured using 12-item grit scale [31] (e.g., “I have overcome setback to conquer an important challenge”) with responses provided on four-point scales (1 = *not like me at all* and 4 = *very much like me*). The scale has demonstrated adequate construct and predictive validity in previous research in school contexts [52].

##### Self-discipline

Students’ self-discipline will be measured using the 10-item self-discipline scale (e.g., “I tend to carry out my plans”) from the IPIP-HEXACO scales [32]. Responses will be provided on four-point scales (1 = *not like me at all* and 4 = *very much like me*). Research has demonstrated the reliability and predictive validity of this scale in school contexts [52].

##### Perceived parental affection and control from parents

Students’ self-reports of their parents’ or legal guardians’ provision of affection, behavioural control and, psychological control will be measured using three scales taken from the modified version [29] of the child rearing practices report (CRPR) [53]: the seven-item parental affection scale (e.g., “My mother/father/legal guardian respects my opinions”), the six-item parent behavioural control scale (e.g., “When my mother/father/legal guardian gets angry, (s)he also shows it”), and the four-item parent psychological control scale (e.g., “My mother/father/legal guardian often reminds me of all the things, (s)he has done for me”). Responses will be provided on seven-point scales (1 = *not at all true* and 7 = *completely true*). Previous research has supported the construct validity and reliability of the scales [54].

##### Perceived autonomy support by parents (or legal guardians) and peers towards out-of-school physical activity

Students’ perceptions of autonomy support from their parents (or legal guardians) and peers will be measured using a four-item scale (e.g., “I feel that my parent(s)/guardian(s)/peers offer(s) me with choices, options, and opportunities to do active sports and/or vigorous exercise”) based on the PASSES [44]. Responses will be provided on 7-point scales (1 = *strongly disagree* and 7 = *strongly agree*). The measure has demonstrated adequate reliability [45].

#### Teachers’ measures

##### PE teachers’ provision of autononomy support and control

Teachers’ self-report of their provision of autonomy support to students in PE lessons will be measured on an adapted six-item version of PASSES (e.g. “I feel that I provide choices and options to my physical education students”) [44]. We also developed an additional item for autonomy support scale to assess teachers’ self-reported provision of autonomy support for student’s participation in leisure time physical activity (“I encourage my PE students to think about how physical activity during PE class can be useful to them during their free time physical activity”) and provision of a rationale for students’ participation in PE (“I feel that I provide choices and options to my physical education students”). Similarly, teachers self-report of their use of controlling behaviours in PE lessons will be measured using an adapted three-item version of the teacher social context questionnaire (e.g., “I always have to tell my PE students what to do”) [55]. Satisfactory psychometric properties have been reported for the original versions of both measures [44, 55]. Responses to items from both scales will be provided on seven-point scales (1 = *completely disagree* and 7 = *completely agree*).

#### Parents’ measures

##### Parental affection, behavioural control, and psychological control

Parents’ or legal guardians’ perceptions of their provision of affection-, behavioural control-, and psychological control towards their child will be measured using three scales [29]: the seven-item parental affection scale (e.g., “I respect my child’s opinions”), the six-item parental behavioural control scale (e.g., “When I am angry at my child, I let him/her know about it”), and the four-item parental psychological control scale (e.g., “My child should be aware of how much I sacrifice for him/her”). Responses will be provided on five-point scales (1 = *not like me at all* and 5 = *very much like me*). The scales have exhibited satisfactory psychometric properties in previous research [29].

##### Parental provision of autonomy support towards out-of-school physical activity

Parents’ or legal guardians’ perception of their provision of autonomy support towards out-of-school physical activity will be measured using a four-item scale (e.g., “I encourage my child to be physically active in free-time”) based on the PASSES [44]. Responses will be provided on 7-point scales (1 = *strongly disagree* and 7 = *strongly agree*).

#### Demographic variables

We will also ask participating PE teachers to self-report the following demographic details: age, gender, education, years of teaching experience, and number of students in their PE class. In addition, we will collect self-reported demographic details from participating students: age, grade, gender, and school. We will also collect the following demographic details from participating parents: gender, nationality of a child, ethnicity of a child, and highest level of education. A summary of study measures, data collection occasions and methods is provided in Additional file 1.

### Sample size

A statistical power analysis was conducted to estimate the required sample size for student data based on a path analysis according to published recommendations [56]. The analysis was based on a model in which the primary outcome variable of student’s participation at each post-intervention follow-up occasion was regressed on the intervention condition (dummy coded as 1 = received intervention, 0 = received control) and constructs from the trans-contextual model (perceived autonomy support, autonomous motivation in PE and out-of-school, attitudes, subjective norms, perceived behavioural control, and intentions) as simultaneous predictors. Statistical power (beta) was set at 0.90 and statistical significance level (alpha) was set at 0.05, and confidence intervals of 0.068 and 0.080 for the root mean square error of approximation fit index based on previous trans-contextual models [9]. The analysis indicated that a student sample size of 286 is required to detect the effect size based on model fit. Based on typical attrition rates of 40% reported in the literature in multiple follow-up studies of physical activity [57, 58] we aim to recruit 476 student participants at baseline (*n* = 238 participants per intervention group).

### Randomization

The core research team will enroll PE teachers and their students to the trial. Schools (*N* = 11) consenting to participate in the trial will be randomized to the intervention or waitlist control conditions. Randomization will be conducted by a researcher independent of the core research team using a random number generator. After generation of the random allocation sequence, the researcher will seal the names of the schools and their allocation in envelopes. Recruitment of teachers and students for the intervention and wait-list control groups will be drawn from the appropriate clusters. The cluster-randomized design precludes potential for contamination of data across conditions caused by the presence of participants from different conditions within schools. The waitlist-control design ensures that the benefits of a potentially effective intervention are not withheld from control group participants.

### Blinding

The researcher who will conduct the randomization of schools to intervention conditions, and the research assistants who will code the audio-recordings of PE teachers’ lessons at baseline and follow-up time points will be blind to group allocation.

### Data analysis

Multilevel structural equation modelling using the Mplus, v. 8.0 software [59] will be used to test our hypotheses. All analyses will be performed using intention-to-treat analysis and supplemented by per-protocol analyses for all planned outcome variables [60]. Where data is missing for the psychological variables, we will impute missing values using linear interpolation if the data is confirmed missing completely at random. We expect to have data on our primary and secondary outcome measures, as well as mediating measures, at pre-trial and at the allotted follow-up occasions after the delivery of the intervention (post-trial, and at the one-, three-, and six-month follow up data collection occasions after the implementation period). We also expect to have self-report data on parenting from students and their parents or guardians at pre-trial. Student data will be nested within school and teacher/class, and therefore variance in outcome variables may be attributable to school-level and class-level variation as well as variation between students attributable to the intervention itself. Effects of the intervention on study outcomes can be interpreted at the student level after controlling for school- and class-level effects. Our longitudinal design enables also the examination of potential trajectories in the development of outcome variables. We will test the model at each of the follow-up time points with students’ out-of-school physical activity as the primary dependent variable, the intervention condition as a dummy-coded independent variable (1 = intervention group; 0 = control group), and the psychological variables (perceived autonomy support from teachers, autonomous motivation in PE and out-of-school physical activity, attitudes, subjective norms, perceived behavioural control, intentions), as simultaneous predictors. We will statistically control for each of model variables from the previous time point using the standardized residual scores.

### Monitoring and intervention adherence plan

The project is led by the core research team comprising the principal investigators, lead researchers, and a doctoral student. The project team is advised by a steering group comprising the core research team and stakeholders. The core research team holds regular meetings to monitor study progress. During intervention planning and development, two meetings with stakeholders, comprising a school PE teacher, a representative of the teacher union, the head of the in-service PE teacher training program, and the head of the city Education Department, will take place to maximize acceptability and adherence to the trial. In addition, the project team will hold regular meetings with stakeholders to discuss content and administration of the intervention. The PE teacher trainers who will deliver the autonomy support training for teachers in the intervention group will receive a familiarization session with the research team during which they will receive instruction on the intervention aims and training materials. They will also have the opportunity to discuss the rationale and expected outcomes of the program to maximize quality of delivery. To increase responsiveness and engagement of the PE teachers, we will use several motivational techniques such as facilitating their autonomous goals or outcomes and clarifying expectations. We aim to offer concrete, clear, and relevant feedback during the teacher training sessions. In terms of intervention fidelity [61, 62], the observation of PE teachers’ lessons during the course of the intervention will serve as a means to ensure fidelity of the intervention delivery to students during the implementation period. In cases where lower than expected compliance with the intervention is identified indicating problems with fidelity, we will create a dichotomous variable representing compliance with intervention and include it as a control variable in our path analyses to test whether fidelity had a significant effect on changes in out-of-school physical activity and other outcomes. Finally, we will content analyse the teachers self-evaluation forms completed after the teacher training period, and the infographic posters produced by each teacher during training that are designed to summarize their learning. These will serve as means to ensure fidelity of the intervention training delivery to participating PE teachers.

### Data management

The University of Jyväskylä will own the research data. Consent forms and paper questionnaires will be stored in locked cabinets in the lead project researcher’s office. Digital data will be stored on password-protected centrally-managed cloud-based storage drives of the Information Management Center at the University of Jyväskylä. All datasets will be de-identified with participants allocated a unique code number. Data files will be managed by core research team members appointed to this task. The key used to identify participants’ data will be stored separately from data files and will only be accessible to designated members of the core research team members. Results will be reported in articles published in established international scientific journals and presentations in scientific and professional congresses. The researchers will target open access publishing and comply with the University of Jyväskylä recommendation of parallel publishing in the University open access digital repository. Results will also be communicated through traditional and social media for the public.

## Discussion

School PE is an existing network with considerable potential for the delivery of interventions to promote physical activity to a captive audience of young people. Such interventions are also consistent with PE curricula to promote lifelong physical activity and health. However, relatively few studies have examined the effectiveness of school-based PE interventions in promoting out-of-school physical activity. The goal of the proposed study outlined in this protocol is to address this gap in the literature by testing the effectiveness of a theory-based intervention delivered in PE to promote lower secondary school students’ physical activity outside of school. The intervention will capitalize on the trans-contextual model [6, 10], a motivation model which specifies the processes by which teachers’ support for students’ self-determined or *autonomous* motivation in school translates to their autonomous motivation, beliefs, and intention toward, and actual participation in, physical activity outside of school.

The intervention will make a unique contribution to knowledge in four areas: (i) it will test the effectiveness of a theory-based in-school intervention delivered by PE teachers in promoting lower secondary school students’ physical activity participation outside of school, which has seldom been demonstrated; (ii) it will evaluate how the intervention works in promoting students’ out-of-school physical activity participation through effects of intervention on key constructs from the trans-contextual model; (iii) it will outline the development and implementation of a cost-effective, replicable theory-based teacher training program to train teachers to use autonomy-support techniques in their PE lessons and promote out-of-school physical activity; and (iv) it will evaluate the long-term effectiveness of the intervention in promoting physical activity behaviour through one, three, and six-month post-intervention follow-up of behavioural and theory-based outcomes.

Although school PE has been identified as an important existing network in which messages and interventions promoting out-of-school physical activity participation could be promulgated, interventions to promote out-of-school physical activity through school PE are rare and these studies often only have limited follow-up periods of behavioural outcomes. For example, a previous study [63] demonstrated the effectiveness of a PE delivered intervention aimed at promoting physical activity participation in high school students. However, the study adopted a relatively brief intervention, relied exclusively on self-reported physical activity measures, and only adopted a relatively short term follow-up of students’ behaviour. Our proposed intervention will advance this research by developing a comprehensive autonomy support training program with an associated set of training materials, use accelerometry to verify self-report measures of physical activity, and conduct a longer-term follow up of intervention effects.

One of the key strengths of the current study is the theoretical basis and the provision to test the mechanisms of effectiveness of the intervention. The current intervention is based on the trans-contextual model [10], which provides a clear basis for the mechanisms of the proposed intervention effects. Specifically, we expect the autonomy-supportive intervention delivered by PE teachers to lead to changes in students’ autonomous motivation in school and outside of school, and their beliefs and intentions toward physical activity outside of school. We will test the effects of the proposed intervention on these theory-based constructs as secondary outcomes in the current intervention. Furthermore, because we plan to collect long-term follow up data we will be able to ascertain whether changes in the psychological constructs as a result of the intervention are maintained over time. The current study is the first to specifically design an intervention using this model and to test the theory-based mechanisms of intervention effects.

One of the potential challenges for the study, raised by external stakeholders, is that Finnish PE teachers are likely to be relatively autonomy-supportive at baseline. Autonomy-supportive teaching is currently emphasized both in the Finnish national PE curriculum and in the Finnish PE teacher training curriculum [7]. This raises the possibility that intervention effects will not be as strong as interventions in other contexts where autonomy support is not part of teacher training [24]. However, despite teachers’ previous exposure to instruction on how to support students’ autonomy, it is unclear to what extent teachers apply these techniques. For example, it is likely that teachers may use these techniques inconsistently, coupled with other, more controlling techniques. Research has demonstrated that even when teachers use autonomy support techniques, if they also use controlling techniques concurrently, it will undermine students motivation and lead to maladaptive outcomes [64]. The potential for previous exposure to autonomy support training notwithstanding, the current program focuses on training teachers’ to use these strategies with greater intensity, specificity, and consistency, so we expect to see changes in the relevant indicators of autonomy support in students post-intervention. Another potential challenge, raised by external stakeholders and teacher educators, is resistance by PE teachers towards supporting students’ autonomy in PE lessons. A key strategy to deal with this challenge is to convince PE teachers that supporting students’ autonomy should not be equated with independence, and autonomy support does not lead to lack of discipline or ‘chaos’ in the classroom [34].

One of the challenges facing interventionists is difficulty in replicating interventions. This is important given the well-publicized need for high-quality replications of interventions that have demonstrated effects to provide converging evidence for effectiveness across contexts and populations. This endeavor is hampered by poor reporting of intervention protocols and content [65]. A strength of the current study is the provision of complete and detailed intervention materials to maximize transparency and potentials for replication. We will provide open access to the materials for the autonomy support training program including the materials used to train teachers and an accompanying manual providing explicit step-by-step instructions for facilitators to run courses to train teachers in autonomy supportive techniques. In addition, study measures and instruments will also be made available. The materials will be made available on the project website: https://osf.io/s4b2g/.

## Additional file

Additional file 1: PETALS measures. Measures, data collection time points and methods in the PETALS intervention. Overview of measures, data collection time points and methods in the PETALS intervention including main outcome measure, secondary outcome measure, mediating measures, additional measures, teacher measures, parenting measures and demographic measures. (DOCX 17 kb)

## Abbreviation

PE: Physical Education

## Footnotes

1 Full details of the schedule and content of the autonomy support training program with accompanying training materials are available as supplemental materials: https://osf.io/s4b2g/

2 As collecting physical activity participation using accelerometry is costly, time-intensive, and highly burdensome on participants, we will only have sufficient resources to conduct these measures in a subsample of participating students. The study will therefore, not have sufficient statistical power to conduct main analyses for intervention effectiveness using this outcome measure. The purpose of the measure is to provide concurrent validity data for the self-report measure of physical activity, which will be used as the primary outcome variable in the present study.
