# Supplementary material for: Using physical education to promote out-of school physical activity in lower secondary school students – a randomized controlled trial protocol
Source: BMC Public Health. 2019 Feb 6;19:157. doi: 10.1186/s12889-019-6478-x (PMC6364480; doi:10.1186/s12889-019-6478-x)
Supplement: Supplementary file 1 — PETALS measures. Measures, data collection time points and methods in the PETALS intervention. Overview of measures, data collection time points and methods in the PETALS intervention including main outcome measure, secondary outcome measure, mediating measures, additional measures, teacher measures, parenting measures and demographic measures. (DOCX 17 kb) [file 12889_2019_6478_MOESM1_ESM.docx]

***Additional file 1***

Measures, data collection time points and methods in the PETALS intervention

| **Assessment** | **Pre-** | **Post-** | **1m. FU** | **3m. FU** | **6m. FU** |
| --- | --- | --- | --- | --- | --- |
| **Main outcome** | | | | | |
| Out-of-school physical activity ^os^ | SR^1^ | SR^1^ | SR^1^ | SR^1^ | SR^1^ |
| **Secondary outcome** |  |  |  |  |  |
| Physical activity behaviour monitoring by accelerometry ^in os^ | PA^1^ | PA^1^ | - | PA^1^ | PA^1^ |
| **Mediating variables** |  |  |  |  |  |
| Perceived Autonomy Support from PE teacher ^in os^ | SR^1^ | SR^1^ | SR^1^ | SR^1^ | SR^1^ |
| Autonomous Motivation ^in os^ | SR^1^ | SR^1^ | SR^1^ | SR^1^ | SR^1^ |
| Controlled motivation ^in os^ | SR^1^ | SR^1^ | SR^1^ | SR^1^ | SR^1^ |
| Amotivation ^in os^ | SR^1^ | SR^1^ | SR^1^ | SR^1^ | SR^1^ |
| Attitude ^os^ | SR^1^ | SR^1^ | SR^1^ | SR^1^ | SR^1^ |
| Subjective Norm ^os^ | SR^1^ | SR^1^ | SR^1^ | SR^1^ | SR^1^ |
| Perceived behavioural control ^os^ | SR^1^ | SR^1^ | SR^1^ | SR^1^ | SR^1^ |
| Intention ^os^ | SR^1^ | SR^1^ | SR^1^ | SR^1^ | SR^1^ |
| **Additional measures** | | | | | |
| Observation of teacher autonomy support | OB^2^ | OB^2^ | - | OB^2^ | OB^2^ |
| Behavioural automaticity | SR^1^ | - | - | - | - |
| Grit | SR^1^ | - | - | - | - |
| Self-discipline | SR^1^ | - | - | - | - |
| Perceived autonomy support from peers ^os^ | SR^1^ | SR^1^ | SR^1^ | SR^1^ | SR^1^ |
| **Teacher measures** |  |  |  |  |  |
| Provision of autonomy support | SR^2^ | SR^2^ | SR^2^ | SR^2^ | SR^2^ |
| Provision of control | SR^2^ | SR^2^ | SR^2^ | SR^2^ | SR^2^ |
| **Parenting measures** |  |  |  |  |  |
| Parental affection | SR^13^ | - | - | - | - |
| Parental behavioural control | SR^13^ | - | - | - | - |
| Parental psychological control | SR^13^ | - | - | - | - |
| Parental autonomy support ^os^ | SR^13^ | SR^1^ | SR^1^ | SR^1^ | SR^1^ |
| **Demographics** |  |  |  |  |  |
| Age | SR^12^ | - | - | - | - |
| Gender | SR^12^ | - | - | - | - |
| Education | SR^23^ |  |  |  |  |
| Nationality of a child | SR^3^ | - | - | - | - |
| Ethnicity of a child | SR^3^ |  |  |  |  |
| School | SR^12^ | - | - | - | - |
| Grade | SR^1^ | - | - | - | - |
| Years of teaching physical education | SR^2^ | - | - | - | - |
| Number of students in teachers’ PE class | SR^2^ | - | - | - | - |

*Note*: SR = Self-report measure; OB = Data collected by observation; Pre = Pre-trial data collection occasion; Post = 1m. FU = etc etc. For each assessment, the method of data collection is indicated as SR for self-reports, OB for observation, PA for physical activity surveillance; to whom the measure is administered, is indicated as: ^1^ for students, ^2^ for physical education teachers, or ^3^ for parents; context evaluated by measure is indicated as ^IS^ = for in-school; ^OS^ = for out-of-school
